# Editorial: Pandemic influenza vaccine approaches: Current status and future directions

**DOI:** 10.3389/fimmu.2022.980956

**Published:** 2022-08-18

**Authors:** Michael Schotsaert, Rebecca Jane Cox, Corey P. Mallett

**Affiliations:** ^1^ Global Health and Emerging Pathogens Institute, Department of Microbiology, Icahn School of Medicine at Mount Sinai, New York, NY, United States; ^2^ Influenza Centre, Department of Clinical Science, University of Bergen, Bergen, Norway; ^3^ GSK, Rockville, MD, United States

**Keywords:** pandemic, influenza vaccines, emergent influenza viruses, animal reservoir, human history

For over a hundred years, humanity was confronted with recurring pandemics caused by influenza viruses, over-burdening health care systems and disrupting societies worldwide. Since the beginning of the previous century, we have encountered four pandemics caused by H1N1, H2N2, and H3N2 viruses, with the last one in 2009 caused by an H1N1 swine-origin virus. With every pandemic a new influenza virus emerged that started circulating in a human population that was immunologically naive against the emerging virus. This condition allows us to study the response to a new vaccine that targets the emerging virus in the absence of pre-existing neutralizing antibodies that may affect vaccine effectiveness. In this Research Topic, Amdam et al., reported on the effect of vaccination history on the immune response to H1N1pdm09 vaccines in health care workers during the period 2009-2014, and Juvet et al., summarized data obtained in the years following the 2009 H1N1 pandemic on vaccine safety for the rolled-out pandemic influenza vaccines.

There is a constant fear that a new and potentially highly pathogenic influenza virus will make the cross-species jump from the animal reservoir to humans and start a new pandemic. Therefore, it came as a surprise for many of us when the second pandemic of this century was caused by SARS-CoV-2, a coronavirus. We are now over two years into the COVID-19 pandemic, and effective vaccines have saved many lives and allowed us to go from worldwide lockdowns towards less restricted travel. The fast response to this newly emerging coronavirus, also from an animal reservoir, was at least partly possible due to pandemic preparedness guided by our knowledge from previous preclinical and clinical research in the context of influenza virus-host interactions, influenza epidemiology, and influenza vaccine development. Interestingly, due to measures in place like social distancing, masking and creating awareness for respiratory virus transmission during the COVID-19 pandemic, hospitalized cases due to influenza virus dropped drastically (1, 2). With easing of restrictions, more social interaction and international travel are again taking off, and thereby influenza cases are also on the rise. The absence of seasonal influenza in humans observed in the first year of the COVID-19 pandemic due to measures taken worldwide to mitigate the spread of SARS-CoV-2 made it difficult to predict the strains to be included in the vaccine, and antigenic mismatch for at least the H3 hemagglutinin (HA) vaccine component has been reported (3, 4). This highlights the need for further investment in pandemic influenza vaccine approaches, as outlined in the strategic plan for a Universal Influenza Vaccine from the National Institute of Allergy and Infectious Diseases (5). A recurring topic is the search for conserved antigens derived from influenza virus proteins and for strategies to make them immunogenic. Moreover, protection can be provided by immune mechanisms other than antibody-mediated virus neutralization, such as T cells or antibody-mediated cellular toxicity (ADCC). As such, different vaccine candidates based on different platforms are suggested. In this Research Topic, conventional (adjuvanted) inactivated influenza virus vaccines are discussed along with mRNA, DNA, virus like particles, T4 bacteriophage-based, and recombinant protein vaccines. Liu et. al., discussed a strategy based on targeting conserved epitopes in the influenza B HA using an engineered inactivated influenza B virus vaccine to induce antibody-mediated protection that correlates with ADCC. A strategy is also being tested in the clinic for an Influenza A universal vaccine that uses chimeric HA influenza vaccines (6, 7). Del Campo et al., described a recombinant protein-based approach to induce cross-reactive influenza nucleoprotein (NP)-specific CD8+ T cells. The nucleoprotein is a conserved influenza T cell antigen also in humans, and therefore targeting the NP may result in protection against influenza viruses from different subtypes. It was already suggested that infection induced NP-specific CD8+ T cells correlated with protection from influenza re-infection for several decades (8). Inducing them by vaccination, however, remained challenging. Interestingly, this methodology seems to be able to induce lung-resident memory CD8+ T cells by intramuscular vaccination, thereby promoting cellular mediated immunity in the tissue where infection starts, allowing for a faster response upon infection. Neuraminidase (NA) is the second major antigenic determinant on the surface of influenza viruses, and infected cells and antibodies against NA correlate with reduced shedding and shorter symptom duration in humans (9). In this Research Topic, Creytens et al., discussed the potential of NA as a vaccine antigen, and Hansen et al., reported that repeated vaccination with seasonal influenza vaccines can boost and maintain NA-specific humoral immunity health care workers in a five-year longitudinal study. The highly conserved ectodomain of the influenza matrix 2 protein (M2e) has been suggested as a good universal vaccine epitope, and antibodies targeting M2e correlated with protection in preclinical and clinical settings (10, 11). However, following natural infection, M2e-specific antibodies are not highly induced, and M2e is poorly immunogenic if not presented to the immune system with the help of a scaffold (12). Li et al., proposed to use the bacteriophage T4 vaccine platform combined with the M2e antigen as a scalable low-cost solution for producing a universal vaccine. Scalability and fast production are crucial for pandemic vaccines, and DNA- and RNA-based vaccine approaches are very attractive for this reason. Nucleic acid-based platforms hold the advantage that vaccine antigen is produced inside host cells and therefore can be efficiently presented to the immune system in the context of major histocompatibility complex proteins. This typically results in efficient T cell activation, as demonstrated by Andersen et al., Nucleic acid-based vaccines can also be recognized by innate immune receptors, thereby further adjuvanting vaccine responses against the antigen they encode, as discussed by Lee and Ryu. Pandemic vaccines often anticipate zoonotic spillover of (avian) influenza viruses from the animal reservoir. Therefore, H5N1 and H7N9-based pandemic vaccines have been tested in humans. Zhou et al., reported that an adjuvanted H5N1 vaccine can efficiently induce HA- and NA-specific antibodies in healthy volunteers that are protective in a preclinical animal model. Finally, preventing spillover of influenza viruses from the animal reservoir may reduce the risk of future pandemics. The one health approach is based on the concept of controlling viruses with zoonotic potential in animal reservoirs, for example poultry, not only to limit circulation of viruses with pandemic potential in the reservoir but also to prevent spread to humans. Kong et al., described in this Research Topic a virus-like particle supplemented with epitope antigens that can be effectively used to prevent H7N9 avian influenza virus from being shed by experimentally infected chickens.

In summary, *Pandemic Influenza Vaccine Approaches: Current Status and Future Directions* provided an interesting and timely update on the current research activities progressing in a highly relevant area of vaccine development that is part of an overall pandemic preparedness strategy, and the editors appreciated the many excellent contributions by several scientific teams.

## Author contributions

All authors contributed to the preparation of the manuscript and reviewed the final content prior to journal submission.

## Conflict of interest

CM is an employee of the GSK group of companies and reports owning shares in GSK.

The remaining authors declare that the research was conducted in the absence of any commercial or financial relationships that could be construed as a potential conflict of interest.

## Publisher’s note

All claims expressed in this article are solely those of the authors and do not necessarily represent those of their affiliated organizations, or those of the publisher, the editors and the reviewers. Any product that may be evaluated in this article, or claim that may be made by its manufacturer, is not guaranteed or endorsed by the publisher.
